# Pincer Nail Deformity: Clinical Characteristics, Causes, and Managements

**DOI:** 10.1155/2020/2939850

**Published:** 2020-04-13

**Authors:** Chao Huang, Rui Huang, Min Yu, Wenlai Guo, Ying Zhao, Rui Li, Zhe Zhu

**Affiliations:** ^1^Department of Hand Surgery, The Second Hospital of Jilin University, No. 218, Lane Ziqiang, Nanguan District, Changchun City, Jilin 130041, China; ^2^Department of Neurosurgery, The Second Hospital of Jilin University, No. 218, Lane Ziqiang, Nanguan District, Changchun City, Jilin 130041, China; ^3^Department of Anesthesiology, Chungking General Hospital, 69 Xingguang Avenue, Yubei District, Chongqing 401121, China

## Abstract

A pincer nail is a common nail deformity of toenails and is characterized by nail thickening and nail plate deformation. It often causes severe pain for patients. We perform a thorough literature review and an additional review of pertinent clinical cases, aiming to provide a comprehensive review of the etiology, pathogenesis, clinical classification, differential diagnosis, and treatment of pincer nail deformity (PND). Understanding the clinical characteristics and treatment progress of a pincer nail will provide clinicians with comprehensive and evidence-based information about PND, thus allowing the selection of an appropriate treatment according to the patient's request and the clinical manifestations of PND, which should maximize patient satisfaction.

## 1. Introduction

Pincer nail deformity (PND) is a common nail deformity first named “incurvated nail” in 1950 by Frost [1] and later as “pincer nail” by Cornelius and Shelley in 1968 [2]. The term pincer nail is generally preferred in recent literature. It has an incidence rate of approximately 0.9% and commonly affects the outside/inside/bilateral sides of the hallux toenails; other toenails and fingernails are infrequently affected [3]. PND is characterized by nail thickening and narrowing of the distal end of the nail bed along the longitudinal axis of the nail plate in a proximal to distal manner together with an increase in the maximum transverse curvature, which causes the nail edges to descend into the lateral nail fold. The curvature that increases along the nail's distal sides causes severe pain, chronic inflammation, and recurrent infections, affecting the daily lives of afflicted patients. PND affects the ability of patients to walk or wear shoes [2], and some severe cases require elective toe amputation [4].

## 2. Etiology and Pathogenesis

### 2.1. Etiology of Pincer Nail

PND's cause is not known but is suspected to be hereditary or acquired. In 1973, Chapman reported three generations of patients with hereditary pincer nails that occurred in their teens and early twenties [5]. They proposed that PND was a hereditary disease that often exhibited symmetry and autosomal dominant Mendelian characteristics [5]. Similar cases were later reported by El-Gammal and Altmeyer, in which the toenails and fingernails of one woman were affected beginning in early childhood [6]. In 2015, Hu et al. observed a multiple-generational Taiwanese family in which approximately half of the family members exhibited PND and symmetry deformities [7]. They also proposed that PND without nail thickening was the main indicator for Clouston syndrome, an autosomal dominant genetic disease.

Unlike hereditary pincer nails, acquired pincer nails exhibit asymmetry. Many systemic diseases are associated with PND, including gastrointestinal malignancies, renal failure, Kawasaki disease, amyotrophic lateral sclerosis, and systemic lupus erythematosus [8, 9]. However, when these diseases are effectively controlled, pincer nails resolve spontaneously. In 1996, Baran suggested that unfitting shoes cause acquired PND [10]. Long-term use of beta-blockers also causes PND, occurring after 6-12 months. However, spontaneous improvement is noticed after stopping the drug. In addition to beta-blockers, pamidronate is another drug suspected to cause PND.

PND is a recognized iatrogenic complication after placement of the arteriovenous fistula (AVF) in the hemodialysis pathway. The pathogenesis involves the development of pseudo-Kaposi's sarcoma and venous hypertension, leading to circulatory disturbances in the microvasculature. Eventually, tissues of the five fingers distal to the AVF become hypoxic. In 2015, Clark and Burns reported that PND occurred averagely 2 years after the formation of AVF and gradually disappeared with reversal of the fistula, indicating that local microcirculation changes and resulting ischemia or venous hypertension could cause PND [11]. Other causes include onychomycosis, epidermolysis bullosa simplex, repeated trauma, nail avulsion, tumor of the nail [12], subungual exogenous osteophytes or peripheral pyogenic granuloma, and osteoarthritis of the distal interphalangeal finger joints.

### 2.2. Pathogenesis of Pincer Nail

Although many cases of hereditary and acquired PND have been reported, the underlying pathogenesis is not known. In 2001, Baran et al. reported that the overcurvature was probably due to exostoses of the distal phalanx, leading to increased torque in the outgrowing nail plate [13]. Recent studies suggested that an osteophyte of the distal phalanx is not a cause but rather a result of nail overcurvature, and the ventral and dorsal side nail plate differences cause PND, through nail bed shrinkage [14]. Similarly, Twigg et al. considered PND to be caused by enlargement of the base of the distal phalanx [15]. The increased tissue leads to a reduced proximal curvature and a larger distal curvature because the nail matrix is firmly attached, resulting in PND.

**Figure 1:**
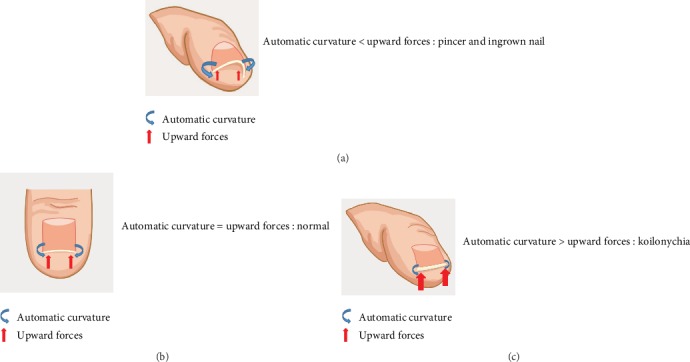
Hypothesis to explain the mechanism underlying the development of nail deformities.

In contrast, some clinicians reported that bedridden patients with no shoes and weight-bearing exhibit a high incidence of developing PND. This suggests that mechanical forces affect the nail formation and pathophysiological process of nail deformity. In 2014, Sano and Ogawa hypothesized that mechanical forces affected the nail configuration and deformation (Figure 1) [16]. Nails naturally bend downward to allow the nail plate to adapt to the daily upward mechanical forces. Under normal conditions, the upward daily mechanical force and downward automatic curvature force are well balanced (Figure 1(b)). However, an imbalance between the two forces may lead to nail deformation. PND is genetically predisposed to the nail bending inward because of the absence of upward mechanical force or increased automatic bending force (Figure 1(a)). Similarly, if the upward mechanical force exceeds the automatic curvature force, the nail will bend outward, forming koilonychias (Figure 1(c)) [16].

## 3. Clinical Classification and Assessment Methods

### 3.1. Clinical Classification of Pincer Nail

PND has three types, as proposed by Baran et al., including the “common” pincer nail (omega or trumpet type, type 1), the plicated nail (similar to the ingrown nail, type 2), and the tile-shaped nail (type 3) [13]. Type 1 is characterized by an increase in the transverse curvature from the proximal to the distal nail, which forms an omega or trumpet shape. Type 2 presents with lateral edges that are sharply bent to form vertical sheets pressing into the lateral nail groove and producing granulation tissues that mimic an ingrown nail. Type 3 is rare and characterized by a larger increase in the transverse curvature along the longitudinal axis of the nail plate, forming a tile shape.

### 3.2. Evaluation Method

**Figure 2:**
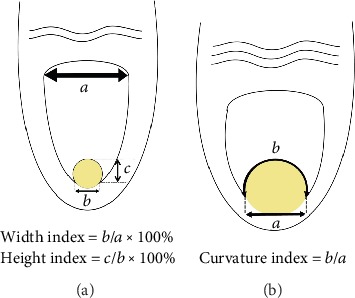
(a) Two indices were used to objectively assess the severity of nail deformities. (b) The curvature index was defined as *b* divided by *a* (*b*/*a*), in which the apparent width of the nail tip was defined as *a* and the traced length of the nail tip was defined as *b*.

Classifying PND severity is complicated, and various terms, such as incurved nail, pincer nail, trumpet nail, and omega nail, have been used to express severity though they were not clearly defined. Moreover, vague results have been reported for the effectiveness of various pincer nail treatments, and no accurate evaluation method exists. Thus, to better assess the severity of pincer nails, Masaaki and Hiroshi in 2003 first developed an original morphological evaluation method that included measuring the width, height, and length of the nail plate [17]. From these data, the following indices were calculated: (1) Width index: width of the nail tip/width of the nail root × 100%. Values closer to 100% indicate wider nails, whereas values approaching 0% indicate more tapered nails. (2) Height index: height of the nail tip/width of the nail tip × 100%. Similarly, values approaching 0% indicate flatter nails, whereas higher values indicate nails displaying a more marked dorsal protrusion. The two indices were used to objectively assess the severity of nail deformities (Figure 2(a)).

In 2013, Yabe proposed a better measurement of the width index than Masaaki and Hiroshi's method [17], which did not account for both side nail roots buried subcutaneously [18]. When the nail's shape is curved, the height index cannot correctly evaluate severity because both the width and the height are reduced. As a result, they proposed a new evaluation system, the curvature index, defined as *b* divided by *a* (*b*/*a*), where the width of the nail tip is *a* and the traced length of the nail tip is *b* (Figure 2(b)). This method allows describing the pincer nail severity and examining clinical treatment progress. However, this evaluation system has some drawbacks including when both nail sides are cut or buried in the lateral nail fold, measurement becomes impossible. Consequently, it is important to develop a more effective evaluation system.

## 4. Differential Diagnosis

The differences between pincer nails and ingrown nails are confusing because they are clinically related; hence, differential diagnoses are required. Ingrown nails are identified based on symptoms, while pincer nails are identified based on morphology. From the morphological perspective, the largest difference between pincer and ingrown nails is that pincer nails show a transverse curvature of the nail plate's long axis that increases in a proximal to distal manner. Moreover, the height of the nail gradually increases, while the nail plate shape of ingrown nails remains normal [19].

## 5. Managements

The aim of PND treatment is to correct the curve that pinches the toes and fingers and yield a cosmetically normal nail [17]. Although conservative, surgical, and combination therapies have been used, there is no standardized PND treatment. Conservative treatment involves a simple operation and is associated with recurrence/temporary remission. Alternatively, the surgical option has a lower recurrence but causes severe pain, poor cosmetic appearance, secondary infection, wound necrosis, and sensory disturbance [20]. Female patients are sensitive to aesthetic problems related to invasive surgery [21]. If acquired PND is accompanied by systemic disease, treating the primary disease improves the symptoms [15]. Antibacterial treatment, nail avulsion, and surgical debridement can treat PND accompanied by secondary infections [4]. Pang et al. reported that PND accompanied by chronic inflammation or recurrent suppurative infection requires regular X-ray examinations to detect early potential osteomyelitis for early intervention [4].

### 5.1. Conservative Treatment (Table 1)

Conservative treatment comprises of auxiliary stents and grinding tools to change the upward mechanical daily force, thickness and growth direction of the nail plate.

#### 5.1.1. Plastic Devices

**Figure 3:**
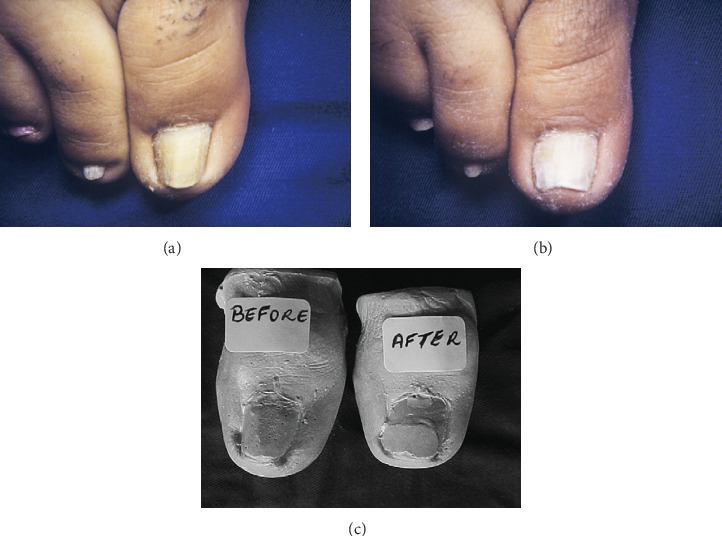
(a) Before treatment. (b) Six months after treatment. (c) Plaster molds before and after treatment [23].

The “clyp system” is a semirigid, flexible plastic material in an elliptical shape. When subjected to pressure, they are malleable [23]. In 1993, Effendy et al. used pliant plastic braces to treat PND in the halluces of three women [22]. In 2006, Nilton et al. used the plaster mold to follow the nail widening during and after treatment to evaluate its efficacy (Figure 3) [23]. Similar to Effendy et al., after slight grinding of the nail plate with sandpaper, the nail surface was degreased with acetone to increase its adhesiveness to the plastic device. A suitable plastic device was then fixed to the nail plate with liquid cyanoacrylate glue. The larger axis of the plastic device matched the horizontal axis of the nail and was close to the free edge. During a follow-up, an alginate mold was applied to the affected hallux. Then, the mold was filled with stone plaster and removed the next day, and the caliper on the plaster mold measured the distance between the lateral nail sides. The biggest drawback for this method is the lengthened treatment period and frequent adjustments of the plastic brace [23].

#### 5.1.2. The Shape-Memory Alloy

**Figure 4:**
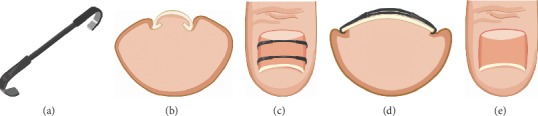
(a) Close-up photograph of the shape-memory alloy device, a central rod made of nickel-titanium and bilateral hooks [31]. (b–e) Serial clinical photographs and objective indices [14].

The shape-memory alloy has a nickel-titanium central rod and bilateral hooks that engage the nail sides (Figure 4(a)) [31]. The central bar is flexible at <25°C, making it easy to bend and apply to severely deformed nails. At >25°C, the bar's solidity increases which corrects the nails. Considering that the foot temperature is approximately 27°C to 28°C, the bar retains its firm nature [24]. In 2009, Kim and Park applied the shape-memory alloy device to treat severely incurved symptomatic toenails and achieved positive outcomes [24]. From 2010 to 2012, they used it on 14 patients with mild and severe PND and achieved satisfactory results (Figures 4(b) and 4(e)). They used a mosquito clamp to remove part of the nail embedded into the periungual skin. One side of a hook was applied to one side of the nail, while the other side of the hook was applied to the opposite side (Figures 4(c) and 4(d)). The hook was moved to the position on the nail corresponding to the starting point of the nail deformity. Finally, one/two similar devices were used depending on toenail size and PND severity. The device was removed after 10 days but can stay for 2 or 3 weeks depending on the toenail changes [14]. Thereafter, in 2014, Lee et al. used this treatment for a single case, and 4 weeks after surgery, skin necrosis developed on the back of the toe [25]. They concluded that it was caused by local infection from self-adherent bandages. Furthermore, the patient had ischemia in the submuscular course of the first dorsal metatarsal artery (FDMA) which may have contributed to necrosis. They concluded that educating the patient on skincare routine minimizes complications. Although Young Joo et al. considered the shape-memory alloy device to be inconvenient [20], the technique is used to improve the PND symptoms [24]. This technique was combined with surgical procedures on patients with hypertrophic nail fold skin [26].

#### 5.1.3. Nail Grinding

**Figure 5:**
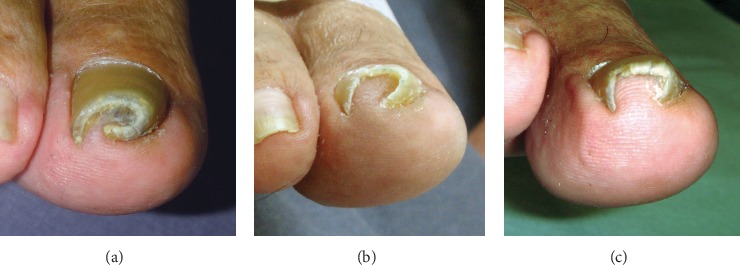
(a) Appearance before treatment. (b) Ten days after nail thinning, the nail showed signs of improvement. (c) Appearance 2 months after nail thinning commenced [28].

Nail grinding is a noninvasive technique, used to treat PND since 1990 [27]. From early studies, Sano and Ichioka reported that the toenails of PND patients were approximately 0.8 mm thicker and stronger bending than those of healthy adults [32]. In 2015, they reported a case of severe PND treated by nail grinding (Figure 5), thereby reducing the automatic curvature force of the nail which balanced the mechanical force and automatic curvature [28]. This technology is evidence that mechanical stimulus-based treatments are effective. Nail deformities can be treated by balancing the automatic curvature force of the nail and the upward mechanical force from the finger/toe pad. Moreover, they suggested that further research is needed to determine the long-term results of this treatment and to establish an optimal and effective thinning method. In addition, massage, machine stimulation, and adjusting the walking posture can be effective. The nail can be softened or thinned using an external preparation, such as 40% urea paste [6] or 3% salicylic acid [29], which reduces the hardness and thickness of nails.

#### 5.1.4. Superelastic Nickel-Titanium

**Figure 6:**
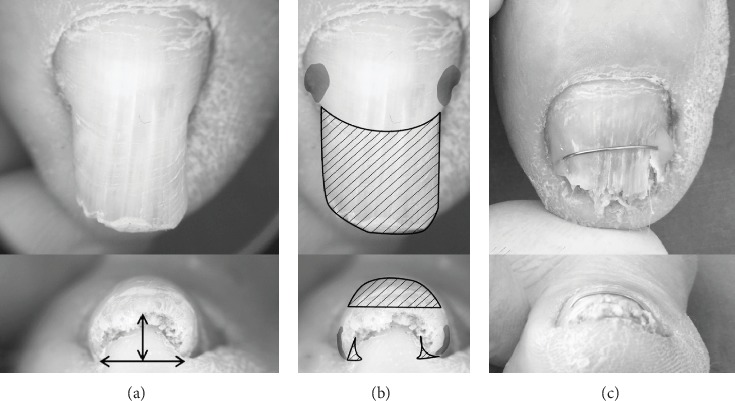
(a) The nail height and width are marked with arrows (height to width ratio 0.833). (b) The hatched area was ground and removed. The gray highlighted portion was slightly ground and bonded with 0.012-inch thick superelastic nickel-titanium wire. (c) One month later, the height to width ratio was 0.25 and the wire was still maintained on the plate [30].

From previous reports, superelastic nickel-titanium enabled bending of ingrown nails to their normal shape, providing a low-cost and effective treatment. Recently, Won et al. used dental correction principles and superelastic nickel-titanium to treat PNDs (Figure 6) [30]. This approach has three advantages. First, it can be tailored to the patient's needs. Second, it can be performed on the nail of the hallux, other toenails, and even fingernails. Third, it is noninvasive and does not limit the patient's lifestyle, enabling prolonged treatment in patients with frequently recurring PND without compliance issues.

### 5.2. Surgical Treatment

In the case of PND with exogenous osteophytes of the distal phalanx or severe dorsal hyperosteogeny, the removal of osteophytes is important [33]. However, for those with no severe bony deformity, operation of the distal phalanx is not necessary [34]. Among the treatments suggested previously, several surgical procedures can be divided into 2 groups: surgical procedures that destroy the nail matrix and those that preserve the nail matrix (Table 2).

#### 5.2.1. Skin Grafts and Other Tissue Grafts

In 1950, Zadik reported this technique for the treatment of ingrown and pincer nails [35]. After nail avulsion, the matrix and epithelium of the posterior wall of the nail were excised completely, and the posterior nail wall was sutured without tension to the nail bed, functioning as an advancement flap. However, two cases of flap necrosis occurred due to flap suturing under tension. In 2004, Iida and Ohsumi introduced a modified version of Zadik's method [36]. After removal of the nail matrix, including approximately 3 mm of the nail bed connected to the distal edge of the matrix, artificial skin material was used to cover the nail bed instead of an advancement flap. It was not successful because of poor wound healing. In both cases, the procedures involved destroying the nail matrix, and the final results were not cosmetically/functionally satisfying. Many patients requested preservation of the nail unit and a good cosmetic outcome. Consequently, most surgeries are aimed at preserving the nail matrix.

In 1979, Suzuki et al. first reported a PND surgical procedure with preservation of the nail matrix [37]. After removal of the nail, a median longitudinal incision was created in the nail bed to spread the tissue on the medial and lateral sides, leaving a center triangular defect. Next, a split-thickness skin graft from the forearm was sutured to this defect. During a follow up, the nail did not adhere properly and became detached. In 2000, Brown and Zook reported a long-term correction of PND by surgically implanting a dermal graft taken from the groin into the affected distal phalanx and nail bed to restore the nail bed contour [38]. However, this method was not positive due to the occurrence of full-thickness skin graft shrinkage [39].

To reduce the floating nail and shrinkage of the graft, Hatoko et al. described the use of a hard-palate mucosal graft to correct severe PND in 2003 [39]. After nail avulsion, an incision was created along the nail bed, and the nail bed tissue flap was raised at the layer above the digital bone. After flattening the digital phalanx, the surrounding tissue was bluntly dissected to spread the shrunken nail bed. When the nail bed tissue returned to its original position, the nail bed defect mainly occurred at the distal end of the bed, and the hard-palate mucosa containing the periosteum was transplanted onto that defect. During the long-term follow-up, no deformity appeared on either side of the nail bed, and epithelial formation occurred spontaneously. Therefore, hard-palate mucosal grafts are an effective option for the treatment of nail bed repair in patients with severe pincer deformities.

#### 5.2.2. Widening of the Nail Bed

*(1) Widening of the Nail Bed with Skin Flap*. In 2003, Masaaki and Hiroshi described a procedure that widened the nail bed in the transverse direction with vertical incisions at the distal end [17]. Sutures were then placed in a zigzag pattern, similar to a classic W-plasty after complete release of the hyponychium and paronychium. Additionally, the skin flap was sutured in a zigzag pattern, which prevented postoperative scar contracture and trapdoor deformity. In 2007, Mutaf et al. [40] reported that removing the osteophytes helps in surgical correction of PND. After the removal of osteophytes on the dorsal surface of the distal phalanx to provide a flat surface for the nail bed, the distal part of the nail bed was enlarged in the transverse direction using a modified 5-flap Z-plasty technique. The 5-flap Z-plasty technique was invented by Mustarde [45] to correct epicanthal folds in 1959. This technique has been used extensively, and the first report of its use to treat PND was described by Mutaf et al. [40].

The above-mentioned authors reported successful results without recurrence, but Cho et al. considered the 5-flap Z-plasty procedure [40] not effective in flattening the nail bed's distal end [20]. They modified the Z-plasty design to improve its ability to flatten the nail bed's distal end and added a vertical incision to the flap to divide the nail bed into two flaps with no vertical skin incision dividing the two Z-designs at the tip of the toes. After flattening the flap (including the raised nail bed), they removed the skin around the two flaps parallel to the nail bed. The transposed flap was sutured with nylon sutures to flatten and widen the nail bed in a transverse direction. Their method utilized the successful techniques from both Masaaki and Hiroshi [17] and Mutaf et al. [40].

*(2) Widening of the Nail Bed with the Combination of Splint and Nail Bed Cutting*. In 2005, a splint made from an aspiration tube was used by Ozawa et al. to correct an elevated periosteal flap with intraoperative compression [21]. They fixed the splint to the proximal nail fold using Schiller's method [46] such that compression of the nail bed onto the distal phalanx and soft tissues was continuous. This technique prevented hematoma formation beneath the periosteal flap, contracture of the nail matrix and nail bed, and direct adhesion of the gauze to the nail bed. Moreover, the nail bed can be monitored for possible infection and flap necrosis using the transparent aspiration tube.

Ghaffarpour et al. proposed that PND arises from the nail bed [41]. Thus, they combined a splint made of an aspiration tube and nail bed cutting to treat PND. After removal of the nail plate, the nail bed was elevated by a “U-shaped” incision consisting of two incisions near the lateral nail and an incision through the distal part of the nail bed and pulp. Next, they created four incisions from the distal part of the lunula to the proximal end of the nail bed parallel to the normal lateral nail grooves. These four incisions ended within 2-3 mm of the distal flap, which made the nail bed wider and stretchable. The lateral parts of this stretchable nail bed were sutured to the lateral skin of the toe, and the distal part was sutured to the tip of the toe. A prefabricated transparent splint with a suction tube in the shape of a normal nail plate was then placed under the proximal nail fold on the nail bed and sutured to the outer nail wall, with the transparent aspiration tube allowing the clinicians to monitor any infection and flap necrosis [21].

#### 5.2.3. Laser Surgery

**Figure 7:**
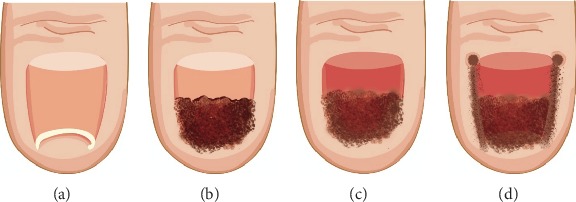
(a) Pincer nail deformity preoperatively. (b) Pincer nail deformity intraoperatively after partial avulsion. (c) Pincer nail deformity intraoperatively after complete avulsion. (d) Pincer nail deformity intraoperatively after complete avulsion and partial matricectomy [34].

Laser treatment is beneficial over surgical excision because it is easy to use, minimalized postoperative care, and has rapid treatment time and minimal pain. Laser treatment is cosmetically friendly, as opposed to the inevitable linear scarring from surgery. In 1988, Leshin and Whitaker described the use of a carbon dioxide (CO_2_) laser for permanent nail ablation via matricectomy to treat PND with a success rate of 100% [42]. Later, Lane et al. advanced this method (Figure 7) [34]. After using a CO_2_ laser to ablate the nail plate, they used it again to perform partial matricectomy to retain medial nail growth and prevent lateral nail regrowth (Figure 7(d)). Thus, removal of the lateral nail matrix is essential for treatment. The CO_2_ laser is beneficial because of its inherent hemostatic properties allowing it to operate on highly vascular anatomical regions like the digits. Additionally, it has shallow penetration depth enabling the destruction of desired regions without causing extensive tissue damage to surrounding structures. This results in reduced healing time and desired cosmetic effects. Subsequently, Miller and Levitt reported successful outcomes after using a pulsed dye laser to treat PND with multiple periungual pyogenic granulomas [43].

#### 5.2.4. Nail Plate and Bed Reconstruction

**Figure 8:**
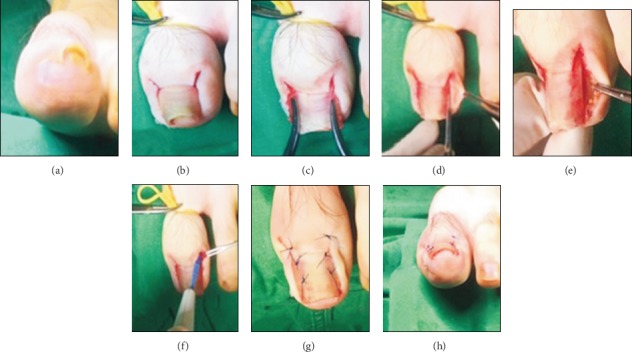
Nail plate and bed reconstruction technique [44].

In 2018, Shin et al. used nail plate and bed reconstruction to treat PND (Figure 8) [44]. They created a 5 mm long incision in the proximal area of the nail along the nail fold to approach the nail matrix (Figures 8(a) and 8(b)). The subperiosteal dissection was performed using an elevator, and the deformed nail plate was removed to prevent injuring the nail bed. Next, using a specific instrument to bend the nail plate at the maximum curve point, the nail bed was gently detached from the distal phalangeal bone using a sharp blade (Figures 8(c) and 8(d)). The area of severe hypertrophy was carefully removed with a small rongeur, and the prominent osteophytes were carefully removed with a small burr. The nail curvature was reevaluated, and the nail bed was flattened. For severe PND cases with lateral deformities or unclear margins, 1-2 mm of the nail was removed from the lateral side of the plate. After resection, any infected lesions were removed (Figure 8(e)). To prevent recurrence, the proximal nail bed was gently ablated (Figure 8(f)). The nail fold was fixed underneath the lifted nail bed to offer support and sutured (Figures 8(g) and 8(h)). This procedure removed the bony osteophytes underneath the nail bed and prevented bony destruction, skin necrosis, and ischemia. Consequently, it is beneficial for severe bony deformities and nail deformities.

### 5.3. Combination Therapy (Table 3)

#### 5.3.1. Nail Plate and Bed Separation Combined with Aluminum Splint Fixation

**Figure 9:**
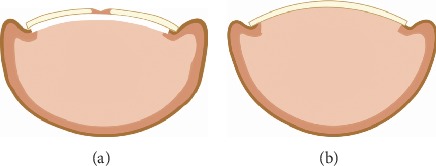
(a) Aluminum splint bar (white color) was glued under the nail plate's white free edge using cyanoacrylate adhesive after nail plate separation [3]. (b) Marked cosmetic improvement 12 months after treatment.

In 2003, Kim and Sim successfully treated 14 patients with severe PND using the nail plate method and bed separation technique combined with aluminum splint fixation (Figure 9) [3]. They used the focused mode of a CO_2_ laser to separate the nail plate by making a longitudinal incision proximally from the lunula border to the distal edge of the nail plate. A longitudinal incision approximately 1 mm in width was created at the center of the curvature to relax the nail plate and straighten it through lifting the edges upward. Next, an aluminum splint bar was attached to the undersurface of the white free edge of the nail plate. The aluminum splint requires a free edge of approximately 2 mm. And it was made from aluminum Nigel splints and was cut to the appropriate size in accordance with the nail size, with a typical size of 3 × 10 × 1 mm. Finally, cyanoacrylate adhesive was applied between the aluminum splint bar and the nail plate, which were bound by needle holders.

#### 5.3.2. Trichloroacetic Acid (TCA) Matricectomy and Aluminum Splint Fixation

Although Kim and Sim successfully treated PND by installing an aluminum splint below the nail plate surface [3], Chi et al. considered it difficult to insert a thick rigid aluminum strip through the gap between the nail plate and the nail bed [47]. They improved the method by fixing an aluminum splint after matricectomy to treat PND. Matricectomy utilizes phenol or sodium hydroxide, although phenol causes systemic side effects, like abdominal pain, hemoglobinuria, and purpura. However, the amount used for matricectomy (<2 ml) is not harmful. TCA, an alternative to phenol, is widely available and safer at concentrations ranging from 9090% to 100%. Both compounds cause coagulative necrosis, but they are safe when used properly [53].

**Figure 10:**
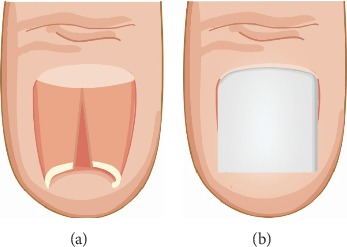
(a) Separation of nail plate using a nail splitter or carbon dioxide laser. (b) Aluminum splint bar fixed over the separated nail plate [47].

TCA use in matricectomy was first reported to treat ingrown toenails. Chi et al. applied it to treat PND [47], using 100% TCA for partial bilateral nail avulsion and matricectomy, with a width of the lateral nail avulsion of approximately 3-4 mm. A septum elevator was then employed to separate the nail plate from the underlying nail bed. Next, a CO_2_ laser or nail separator was used to longitudinally separate the nail plate throughout the lunula to the distal edge of the nail plate (Figure 10(a)). This longitudinal avulsion reduced the curvature of the distal nail plate. Finally, an aluminum splint bar of appropriate size was attached to the nail, and the bar was fixed to the nail plate using a self-adhesive wrap (Figure 10(b)). A satisfactory effect was obtained after a long-term follow-up. In 2015, a successful similar procedure was used by Evgenia et al. [48].

#### 5.3.3. Surgical Matricectomy, Thioglycolic Acid (TGA), and Anticonvex Sutures

**Figure 11:**
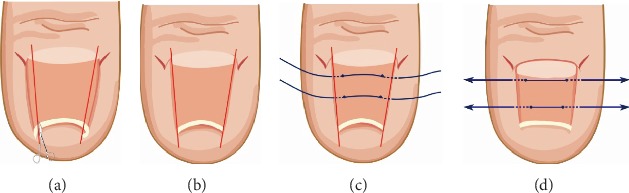
Schematic diagram of the treatment of surgical matricectomy, thioglycolic acid (TGA), and anticonvex sutures [49].

In 2017, Dikmen et al. used surgical matricectomy combined with TGA and anticonvex sutures in a study of 19 cases of PND in 14 patients [49]. They thickly applied a 5% TGA solution, embedded in gauze, directly to the affected toenail surface while the patients were in the preoperative waiting room. The nail was then covered with a minimal dressing to ensure contact between the TGA and nail surface to soften the nail plate. Thirty minutes after application, two small oblique incisions were created on the skin of the lateral aspects of the eponychial fold (Figure 11(a)). The nail plate was cut longitudinally as a nail strip using a straight pair of scissors to an approximate 3/4 mm width. Subsequently, the nail plate's ingrown segment was removed. After raising the eponychial flap outward with a hook, the nail matrix down to the periosteum was exposed and excised (Figure 11(b)). Next, after the 1-0 polypropylene suture, 2 correcting anticonvex sutures were placed in the proximal and distal parts of the softened nail plate to straighten the plate (Figures 11(c) and 11(d)). The anticonvex sutures were removed 3 months later. Using this procedure, there was a cosmetic nail shape and less pain and trauma to surrounding tissues, when compared with those achieved with flap techniques [38, 40].

#### 5.3.4. Nail Methylation Phenolization (NMP) Combined with Surgical Treatment

The NMP has the advantages of an easy surgical procedure without specialized equipment, a minimal surgery time combined with minimal postoperative pain and bed rest, and low recurrence rates. Additionally, because phenol is antiseptic, the NMP technique can be used to treat PND complicated with infection. In early years, this method was extremely effective to treat ingrown nails. Up until 2001, Aksakal et al. used the combination of chemical matricectomy with nail bed repair to correct PND [50]. In 2001, Plusjé also used phenol combined with surgery to correct PND [51]. They applied phenol to the matrix horns before operating on the distal phalanx, contrasting Sugamata and Inuzuka's method [52].

**Figure 12:**
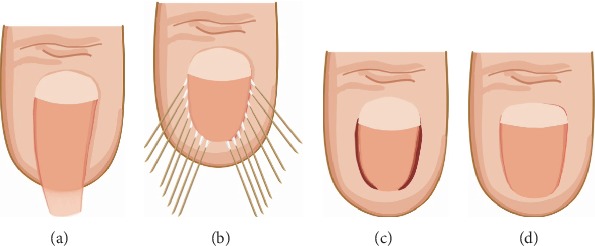
(a) Before the treatment; (b) during the treatment; (c) immediately after the application of phenol; (d) eight months later [52].

In 2011, Sugamata and Inuzuka incised the nail plate longitudinally from the top to the root with fine-tipped scissors [52]. The excised nail's width was approximately 4-5 mm from the lateral edge of the nail plate. The incurved distal third of the nail plate was then excised transversely (Figure 12(a)). A fine cotton-tipped applicator was immersed in phenol solution at a concentration greater than 88% *v*/*v*. The posterior nail fold, nail matrix, nail bed, and lateral nail fold were cauterized completely with 5-6 cotton-tipped applicators that had been immersed in phenol for approximately 5 minutes (Figures 12(b) and 12(c)). The site of cauterization was washed with a sufficient amount of saline to inactivate the residual phenol. As a result, the nails returned to their normal lengths in 2-4 months (Figure 12(d)). All patients reported an appreciable improvement in their PND and the disappearance of pain from the halluces. Additionally, there were no serious complications, such as necrosis or phenol intoxication. The only disadvantage of this approach was the narrowness of the nail.

## 6. Conclusions

The pincer nail is a common nail deformity with a complicated pathogenesis and etiology. Many effective methods for the treatment of PND have been reported, including conservative treatment, surgical treatment, and combination therapy. However, no consensus has been reached regarding the suitable method for correcting PND, necessitating further research. Although many treatments described in the literature have demonstrated good results, these findings may be subject to publication bias and influenced by patient choice. Satisfactorily treating PND is not easy, and an appropriate clinical treatment method should be selected according to the patient's request and the clinical manifestations of PND to maximize patient satisfaction.

## Figures and Tables

**Table 1:** Conservative treatment for PND.

No.	Authors and references	Year	Cases	Age (years)	Sex	Location	Treatment	Complication	Outcome	Limitations
1	Effendy et al. [22]	1993	3	NM	F/3	Halluces	Pliant braces after flattening with a grinder	NM	Good	NM
2	Chiacchio et al. [23]	2006	27	53, average	M/2, F/25	Halluces	Plastic device	No	Good	Longer treatment period; required several plastic brace adjustments
3	Kim and Park [24]	2009	19	38.8, average	NM	Toenails	Shape-memory alloy (the K-D)	6% recurrence rate	Good	NM
4	Kim et al. [14]	2013	21	51.9, average	M/3, F/11	Halluces	Shape-memory alloy device	No	Good	Eczema lesions; sensitivity of the shape-memory alloy device
5	Lee et al. [25]	2014	1	27	F	Hallux	Shape-memory alloy brace	Superficial necrosis	Bad	NM
6	Yang et al. [26]	2011	1	32	M	Halluces	Shape-memory alloy and removal of excess skin and subcutaneous fat	No	Good	NM
7	Roh et al. [27]	1997	1	60	F	Fingers	Nail grinding method, 3 times weekly	No	Good	NM
8	Sano and Ogawa [28]	2015	1	55	M	Left hallux	Reduce the hardness and thickness of nails using a nail grinder	NM	Good	NM
9	El-Gammal and Altmeyer [6]	1993	1	39	F	Most toenails, all fingernails	40% uric acid ointment	NM	Good	NM
10	Baran et al. [29]	2002	NM	NM	NM	NM	3% salicylic acid	NM	Good	NM
11	Won et al. [30]	2018	68	46, average	M/21,F/47	Toenails	Superelastic nickel-titanium	Early wire detachment (4/68), discomfort (2/68), torn stockings (3/68)	Good	NM

NM: not mentioned; F: female; M: male.

**Table 2:** Surgical treatment for PND.

No.	Authors and references	Year	Cases	Age (years)	Sex	Location	Treatment	Complication	Outcome	Limitations
1	Zadik [35]	1950	16	NM	NM	NM	Advancement flap after destroying the nail matrix	Little epithelial thickening over the nail bed; necrosis of the flap	Satisfactory	Permanent nail eradication, loss of fingertip dexterity, and aesthetic differences
2	Iida and Ohsumi [36]	2004	14	67.5, average	M/4, F/10	Halluces, fingers	Modified Zadik method with artificial skin	No	Good	Wound took longer to epithelialize
3	Suzuki et al. [37]	1979	NM	NM	NM	NM	Preserving the nail matrix with a split-thickness skin graft	NM	NM	Nail did not adhere to the split-thickness skin graft, resulting in a floating, distorted nail
4	Brown and Zook [38]	2000	6	52, average	M/1, F/5	Halluces (2/6), thumb (4/6)	Implanting dermal grafts between the distal phalanx and nail bed to restore the nail bed contour	NM	NM	Shrinkage of the full-thickness skin grafts
5	Hatoko et al. [39]	2003	1	25	M	Bilateral halluces	Hard-palate mucosal graft after flattening the digital bone	No	Good	No
6	Masaaki and Hiroshi [17]	2003	27	NM	NM	Halluces (40)	Widening the nail bed with a zigzag flap	No	Good	No
7	Mutaf et al. [40]	2007	8	17 to 48	M/2, F/6	Toenails	Modified 5-flap Z-plasty technique to enlarge the distal part of the nail bed after removing the osteophyte	Infection and partial wound dehiscence (1/8)	Good	Limited ability to flatten the distal end of the nail bed [66]
8	Cho et al. [20]	2015	12	43, average	M/3, F/9	Toenails	Modified double Z-plasty	No	Good	No
9	Ozawa et al. [21]	2005	7	41.5, average	M/2, F/5	Right hallux (4/9), left hallux (1/9), bilateral halluces (4/9)	Splinting device composed of an aspiration tube	Ingrowth of the nail (1/9)	Good	No
10	Ghaffarpour et al. [41]	2010	11	60, average	M/2, F/9	Toenails	Widening the nail bed with the combination of splint and nail bed cutting	No	Good	No
11	Leshin and Whitaker [42]	1988	9	NM	NM	NM	Carbon dioxide (CO_2_) laser for permanent nail ablation via matricectomy	No	Good	No
12	Lane et al. [34]	2004	1	63	M	Left thumbnail	CO_2_ laser to ablate the nail plate and lateral horns of the matrix; performance of a partial matricectomy; satisfactory results were achieved	No	Good	No
13	Miller and Levitt [43]	2011	1	16	M	Left third finger	Pulsed dye laser	No	Good	No
14	Shin et al. [44]	2018	11	61.7, average	M/7, F/4	Halluces	Nail plate and bed reconstruction	Mild ischemic changes on the incision, but with healed wounds (2/11)	Good	No
15	Altun et al. [9]	2016	1	64	F	Right hallux	Removal of osteophytes and correction of the depressed areas of both sides of the nail bed (lateral nail fold) with dermal flaps prepared from the side	No	Good	No
16	Yabe [18]	2013	1	51	F	Right hallux	Removal of the nail plate, raising the nail bed with a periosteum as a flap, flatting the distal phalanx, and trimming excessive skin of both sides of the nail	No	Good	No
17	Fuchsbauer et al. [33]	2007	1	46	M	Right hallux	Removal of the nail plate, elevation of the nail bed, flattening of the distal dorsal bony excrescence, placing a dermal graft, and placing silicon sheeting	No	Good	No
18	Majeski et al. [8]	2005	1	29	F	All fingernails	Resection of the nail plate and matrix	NM	Good	NM
19	Brown and Zook [12]	1988	1	45	F	Right thumb	Removal of the cyst and two corners of the matrix to reduce the width of the nail	No	Good	No

NM: not mentioned; F: female; M: male.

**Table 3:** Combination therapy for PND.

No.	Authors and references	Year	Cases	Age (years)	Sex	Location	Treatment	Complication	Outcome	Limitations
1	Kim and Sim [3]	2003	14	NM	NM	Left hallux	Nail plate and bed separation combined with aluminum splint fixation	No	Good	Insertion of a thick, rigid aluminum strip through the gap between the nail plate and nail bed requires a difficult operation [20]
2	Chi et al. [47]	2010	1	35	M	Bilateral halluces	TCA matricectomy and aluminum splint fixation	No	Good	NM
3	Chi et al. [47]	2010	1	36	M	Bilateral halluces	TCA matricectomy and aluminum splint fixation	No	Good	NM
4	Chi et al. [47]	2010	1	25	M	Bilateral halluces	TCA matricectomy and aluminum splint fixation	No	Good	NM
5	Chi et al. [47]	2010	1	11	M	Bilateral halluces	TCA matricectomy and aluminum splint fixation	No	Good	NM
6	Chi et al. [47]	2010	1	33	F	Right hallux	TCA matricectomy and aluminum splint fixation	No	Good	NM
7	Chi et al. [47]	2010	1	16	M	Left hallux	TCA matricectomy and aluminum splint fixation	No	Good	NM
8	Chi et al. [47]	2010	1	43	M	Bilateral halluces	TCA matricectomy and aluminum splint fixation	No	Good	NM
9	Markeeva et al. [48]	2015	1	65	M	Right thumb	Failure of 40% urea paste, followed by bilateral nail resection, matricectomy with 90% TCA, incision of the median nail, and splinting	No	Good	No
10	Dikmen et al. [49]	2017	14	45.2, average	M/4, F/10	Halluces	Surgical matricectomy, thioglycolic acid, and anticonvex sutures	Superficial infection (1/14), recurrence (1/14)	Satisfactory	Poor cosmetic appearance (15.8%)
11	Aksakal et al. [50]	2001	10	32-47	M/4, F/6	Bilateral halluces (4/10), unilateral toenail (6/10)	Combination of chemical matricectomy with phenol and nail bed repair	Wound oozing for a few weeks	Good	No
12	Plusjé [51]	2001	6	NM	NM	NM	Application of phenol to the matrix horns combined with surgical treatment	NM	Good	NM
13	Sugamata and Inuzuka [52]	2011	9	51, average	M/1, F/8	Halluces (11)	Methylation phenolization combined with surgical treatment	Recurrence (1/11)	Good	Narrow nail

NM: not mentioned; F: female; M: male.
